# Changes in Subway Ridership in Response to COVID-19 in Seoul, South Korea: Implications for Social Distancing

**DOI:** 10.7759/cureus.7668

**Published:** 2020-04-14

**Authors:** Jewel Park

**Affiliations:** 1 College of Medicine, Korea University, Seoul, KOR

**Keywords:** covid-19, social distancing, south korea, public transport, risk perception

## Abstract

Introduction

While numerous episodes of Coronavirus disease 2019 (COVID-19) infection and subsequent government announcements in South Korea were accompanied by widespread social distancing efforts by the people, it is unclear whether these episodes and government announcements were actually influential in improving social distancing, or whether the level of response among different demographic groups varied.

Methods

Data were downloaded from Seoul Data Open Plaza, and changes in the number of passengers on the Seoul Metropolitan Subway network between January 1, 2020, and March 31, 2020, were used to assess the extent to which people in Seoul practiced social distancing. Five events regarding COVID-19 that received wide public attention between January and March 2020 were identified and the changes in the number of passengers before and after each event were analyzed. Also, similar analyses were performed for 16 stations that were specific in either the age or purpose of the visit of the passengers.

Results

Compared to the third week of January 2020 (January 13-19), the mean daily number of passengers in all stations decreased by 2,984,857.4 or 40.6% by the first week of March (March 2-8). The percentage decrease in individual stations between this period was not significantly different between “young” and “old” stations (46.3% vs. 49.2%; p = 0.551) but was significantly smaller in “work” stations than in “leisure” stations (36.2% vs. 51.6%; p = 0.021). Of the five events, the first reported death due to COVID-19 in South Korea and the identification of a mass infection cluster in Daegu on February 20 were accompanied by the greatest decrease of the mean daily number of passengers (1,352,153.3 or 20.8%), while the first mass infection in Seoul on March 10 and the announcement of aggressive social distancing campaign on March 22 were accompanied by an increase in the number of passengers.

Conclusions

The number of subway passengers in Seoul decreased markedly during late February but slowly increased afterward, suggesting decreasing levels of risk perception and adherence to social distancing. Understanding the differing patterns of subway use by age or purpose of the visit may guide policymakers and the general public in shaping their future response to the current pandemic.

## Introduction

Since the outbreak of Coronavirus disease 2019 (COVID-19) in Wuhan, China, the response in South Korea has received widespread attention from the world, as it was able to slow down what was once an explosive outbreak without resorting to forceful measures such as nationwide lockdowns. It is thought that South Korea’s diagnostic capacity, contact tracing, and transparent information, as well as voluntary social distancing, may have been the keys to its success. However, whether that success will be maintained is uncertain [1]. As the outbreak reached its peak on February 29, the Korea Centers for Disease Control and Prevention (KCDC) had encouraged the people to practice social distancing, and although the number of new cases decreased sharply afterward, smaller outbreaks of mass infections continued, and the KCDC accordingly announced a more aggressive social distancing campaign from March 22 [2].

In an emerging epidemic, human behavioral changes are driven by risk perceptions, and the resulting responses can affect the spread of infections [3]. While two online surveys of 1,000 South Koreans in late February and late March released by the KCDC revealed that 75.4% and 88.7% of respondents, respectively, refrained from using public transport, it is not clear whether such high levels of risk perception are representative of the extent to which the general public actually practiced social distancing [4]. It is also important to be aware as to how specific population groups changed their behavior in response to COVID-19, since susceptible groups, such as the elderly, are more likely to progress to severe cases of COVID-19, and since other groups, such as those working in companies, may have less autonomy over practicing social distancing [5,6]. Understanding people’s behavior is crucial in responding to COVID-19 as it forms the basis for changing behavior [7].

The Seoul Metropolitan Subway is the most common form of public transportation used by the people in Seoul, with an average of 13 million boarding and alighting passengers per day. Some of the stations have specific age groups or specific diurnal and weekly patterns of passengers, so the changes in the number of passengers in these stations may provide valuable information regarding the changing behaviors of various population groups [8]. Thus, this study explores the changes in the number of subway passengers in Seoul at various time points of the COVID-19 outbreak in South Korea.

## Materials and methods

Data collection

The number of daily confirmed cases in South Korea and Seoul was obtained from the KCDC website, and the number of daily passengers in the Seoul Metropolitan Subway was obtained from the Seoul Open Data Plaza [9]. The numbers of alighting passengers were collected for 598 stations in 16 lines, i.e., lines 1 to 9, Airport Railroad Express, Gyeongui-Jungang line, Gyeongchun line, Bundang line, Suin line, Gyeonggang line, and Ui-Sinseol line from January 1, 2020, to March 31, 2020. Also, subway data for 2019 was also collected for comparison. Seoul Metropolitan Subway also provides free senior passes to the elderly aged 65 and over, and data for senior pass use rates in 2019 were obtained as well [10].

Classification and characteristics of subway stations

Sixteen subway stations were identified for individual analysis. Each is a major subway station in Seoul that recorded at least five million boarding and alighting passengers in 2019 and has defining characteristics related to factors such as passenger age (“young” or “old”) or purpose of visit (“work” or “leisure”).

“Young” Stations

Among stations that are close to universities, four “young” stations with the lowest senior pass use rates in 2019 were identified: Hongik University station, Konkuk University station, Sinchon station, and Seoul National University station (with senior pass use rates of 4.7%, 7.4%, 10.3%, and 12.9%, respectively). While Hongik University station and Konkuk University station are transit stations, data for only line 2 was used for both stations, as line 2 exits in these stations had lower senior pass use rates. While these stations usually experience an increase in the number of passengers in March as the spring semester starts, most universities in Seoul have resorted to online lectures for March 2020 due to COVID-19.

“Old” Stations

Four “old” stations with the highest senior pass rates in 2019 were identified: Jegidong station, Cheongnyangni station, Jongno 3-ga station, and Jongno 5-ga station (with senior pass use rates of 54.2%, 41.8%, 36.9%, and 34.4%, respectively). While Cheongnyangni station and Jongno 3-ga station are transit stations, data for only line 1 was used for both stations, as line 1 exits in these stations had higher senior pass use rates.

“Work” Stations

Four “work” stations were identified based on their proximity to the main business districts of Seoul and the relative absence of entertainment or residential areas: Gasan Digital Complex station, Yeoksam station, Yeouido station, and City Hall station. These stations have high numbers of alighting passengers in the morning and boarding passengers in the evening on weekdays, which is typical of workplace destinations [8].

“Leisure” Stations

Four “leisure” stations were identified based on the weekly pattern of passengers that peaked on weekends: Express Bus Terminal station, Yeongdeungpo station, Hyehwa station, and Itaewon station [8]. Express Bus Terminal station and Yeongdeungpo station are both major shopping destinations, each directly connected large shopping malls, while Hyehwa station and Itaewon station are famous for nearby venues for theatrical performances and nightlife, respectively. While Express Bus Terminal station is a transit station, data for only line 3 was used, as line 3 exits are directly connected to the shopping mall.

Selection of dates

To observe an overall change in the number of subway passengers, the absolute and percentage changes in the number of passengers were calculated between a reference week (January 13-19) and the week with the minimum number of passengers (March 2-8).

The responses of subway passengers to specific events were also observed. In Seoul, there were five major events between January 2020 and March 2020 that received public attention throughout the outbreak of COVID-19 [11]. As up to four cases were confirmed with COVID-19 in South Korea by January 27, the KCDC raised its infectious disease alert level from “Yellow” to “Orange” in an effort to strengthen the response system in the communities. On February 20, the first death due to COVID-19 in South Korea was reported, and this was accompanied by the identification of mass infection in Daegu, a city approximately 250 km southeast of Seoul. By February 29, the number of new confirmed cases reached a peak with 909 cases. On March 10, mass infection was reported for the first time in Seoul, which had been relatively spared from COVID-19 until this point in time. On March 22, the KCDC started a campaign for aggressive social distancing in an effort to stem the occurrence of new cases. The absolute and percentage changes in the number of passengers seven days after each of these events were compared to those of the day of the event and six days prior. An exception was made for dates between January 28 and February 3, which were compared to seven to 13 days ago, since the Lunar New Year period (January 24-27) and days leading up to it likely caused atypical subway usage.

Statistical analysis

Line graphs for the number of passengers and bar graphs for the number of COVID-19 cases were generated using the “ggplot” package in R. Unpaired t-test was used to calculate p-values for the mean differences in the percentage change of passengers between “young” and “old” stations, or between “work” and “leisure” stations. A p-value of less than 0.05 was considered to be statistically significant. All analyses were performed in R 3.6.3 (The R Foundation for Statistical Computing, Vienna, Austria).

## Results

**Figure 1:**
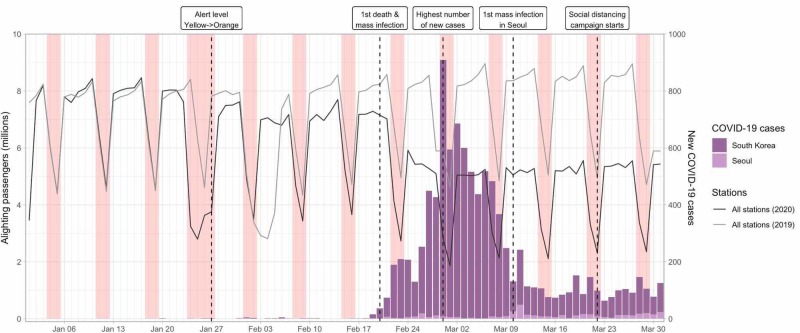
Number of daily alighting passengers for all stations in the Seoul Metropolitan Subway and number of new COVID-19 cases in Seoul and South Korea The number of subway passengers in 2019 was shifted forward by one day to match the days of the week Red bars represent the weekend; the wider bar between January 24 and January 27 represents the Lunar New Year period

**Figure 2:**
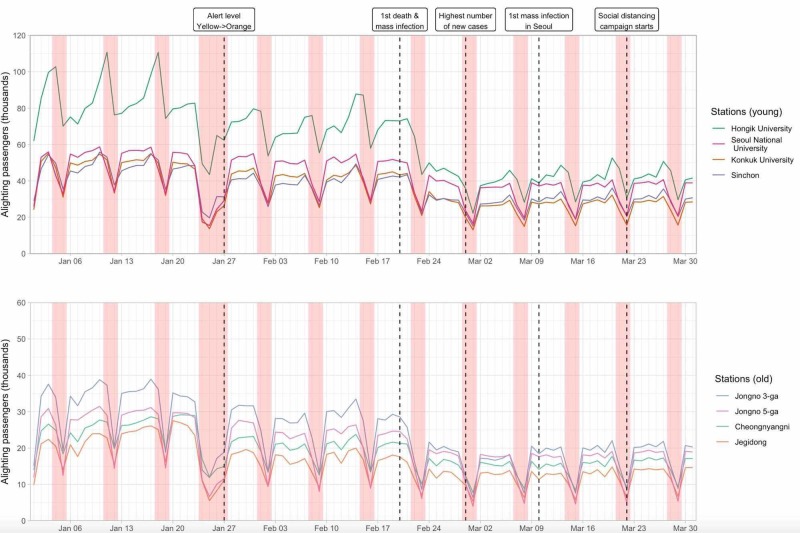
Number of daily alighting passengers for 16 stations in the Seoul Metropolitan Subway classified by the age of passengers Red bars represent the weekend; the wider bar between January 24 and January 27 represents the Lunar New Year period

**Figure 3:**
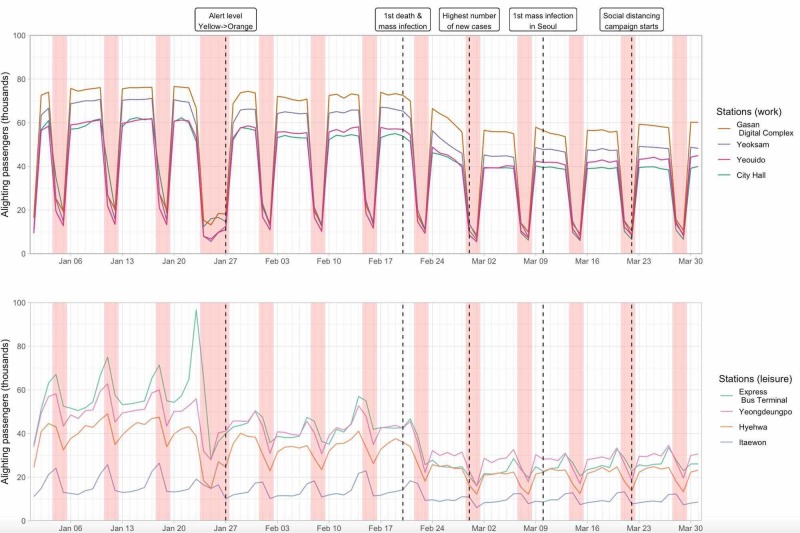
Number of daily alighting passengers for 16 stations in the Seoul Metropolitan Subway classified by purpose of visit Red bars represent the weekend; the wider bar between January 24 and January 27 represents the Lunar New Year period

The changes in the number of passengers in all stations combined and the number of new cases each day in South Korea and Seoul are displayed in Figure 1. The decrease in the number of passengers between January 24 and January 27, 2020, is due to the Lunar New Year period, which is the most important holiday season in Korea. While the numbers in early January 2020 were similar to those of 2019, they decreased slightly after January 27, dropped sharply after February 20, and reached a minimum in the first week of March (March 2-8). The changes for 16 selected stations are also shown, classified by age of passengers or purpose of visit (Figures 2, 3).

Compared to the reference week (January 13-19), the mean daily number of passengers in all stations decreased by 2,984,857.4 or 40.6% by the first week of March (March 2-8) (Table 1). When the changes in the mean daily number of passengers seven days before and after the five important events were calculated, the greatest absolute and percentage decrease for all stations combined was recorded after February 20 (1,352,153.3 or 20.8 %) (Table 2).

**Table 1:** Changes in the mean daily number of passengers between the reference week (January 13-19) and the minimum week (March 2-8)

	Reference week	Minimum week	Absolute change	Percentage change
All stations	7,353,900.0	4,369,042.6	2,984,857.4	-40.6
Young: Hongik University	87,107.1	38,751.7	48,355.4	-55.5
Young: Sinchon	47,339.9	26,708.7	20,631.1	-43.6
Young: Konkuk University	48,642.0	24,546.3	24,095.7	-49.5
Young: Seoul National University	51,829.4	32,966.1	18,863.3	-36.4
Old: Jongno 3-ga	33,776.7	15,216.3	18,560.4	-55.0
Old: Jongno 5-ga	27,785.3	15,023.9	12,761.4	-45.9
Old: Cheongnyangni	26,174.3	14,231.4	11,942.9	-45.6
Old: Jegidong	23,749.9	11,761.4	11,988.4	-50.5
Work: Gasan Digital Complex	61,111.7	43,303.6	17,808.1	-29.1
Work: Yeoksam	56,652.1	34,996.7	21,655.4	-38.2
Work: Yeouido	48,409.1	30,875.0	17,534.1	-36.2
Work: City Hall	51,611.9	30,266.9	21,345.0	-41.4
Leisure: Express Bus Terminal	58,231.9	22,761.3	35,470.6	-60.9
Leisure: Yeongdeungpo	51,883.7	26,783.4	25,100.3	-48.4
Leisure: Hyehwa	42,758.0	19,371.7	23,386.3	-54.7
Leisure: Itaewon	16,884.3	9,689.4	7,194.9	-42.6

**Table 2:** Changes in the mean daily number of passengers between seven days before and after five important events Five important events: January 27 - the KCDC raises its infectious disease alert level from “Yellow” to “Orange”; February 20 - the first death due to COVID-19 in South Korea reported; February 29 - the number of new confirmed cases reaches a peak with 909 cases; March 10 - mass infection reported for the first time in Seoul; March 22 - the KCDC starts a campaign for aggressive social distancing N: the absolute change in the mean daily number of passengers; %: the percentage change in the mean daily number of passengers; KCDC: Korea Centers for Disease Control and Prevention

Stations	January 27		February 20		February 29		March 10		March 22	
	N	%	N	%	N	%	N	%	N	%
All stations	-925,717.9	-12.6	-1,352,153.3	-20.8	-343,574.0	-7.3	62,090.7	1.4	107,398.9	2.3
Young: Hongik University	-16,719.9	-19.1	-21,611.6	-29.1	-6,208.4	-14.1	1,688.3	4.3	131.0	0.3
Young: Sinchon	-9,260.1	-19.5	-9,499.7	-23.2	-1,668.1	-6.0	1,412.9	5.2	373.3	1.3
Young: Konkuk University	-7,431.0	-15.3	-10,247.7	-24.6	-3,146.4	-11.5	791.4	3.2	131.6	0.5
Young: Seoul National University	-4,685.6	-9.0	-8,583.9	-18.3	-2,545.6	-7.3	220.0	0.7	795.0	2.3
Old: Jongno 3-ga	-6,366.1	-18.8	-8,116.6	-30.1	-2,176.3	-12.6	1,283.6	8.1	462.4	2.6
Old: Jongno 5-ga	-4,904.4	-17.6	-4,742.7	-21.9	-789.0	-5.0	-38.3	-0.3	637.4	4.1
Old: Cheongnyangni	-5,906.1	-22.3	-4,296.3	-21.2	-685.3	-4.6	155.6	1.1	605.7	4.1
Old: Jegidong	-7,396.9	-30.5	-3,979.1	-24.1	-18.4	-0.2	37.1	0.3	630.9	5.2
Work: Gasan Digital Complex	-4,483.3	-7.3	-7,222.1	-12.6	-4,404.3	-9.3	-870.9	-2.0	1,746.6	4.0
Work: Yeoksam	-5,739.6	-10.1	-9,701.9	-18.4	-4,832.6	-12.1	1,093.9	3.0	754.0	2.0
Work: Yeouido	-4,229.9	-8.7	-7,922.6	-17.5	-3,786.7	-11.0	644.7	2.0	1,089.9	3.3
Work: City Hall	-7,205.1	-13.9	-7,131.3	-16.2	-4,026.0	-11.7	-231.6	-0.8	124.7	0.4
Leisure: Express Bus Terminal	-14,960.3	-25.6	-15,877.6	-34.3	-2,395.6	-9.7	1,412.1	6.0	743.3	2.9
Leisure: Yeongdeungpo	-8,840.9	-17.0	-11,531.7	-26.3	-2,034.3	-7.1	-861.6	-3.2	1,204.6	4.4
Leisure: Hyehwa	-8,924.0	-20.8	-9,207.7	-26.5	-3,263.0	-14.4	991.0	5.1	381.1	1.8
Leisure: Itaewon	-3,462.3	-20.5	-3,729.1	-24.1	-378.3	-3.9	54.0	0.6	-357.7	-3.6

The decrease in the mean daily number of passengers between the reference week and minimum week was significantly different when stations were classified as “work” and “leisure” (36.2% vs. 51.6%; p = 0.021), but not when classified as “young” and “old” (46.3% vs. 49.2%; p = 0.551) (Table 3). “Work” stations reported a smaller percentage decrease than “leisure” stations after January 27 (10.0% vs. 21.0%; p = 0.003) and February 20 (16.2% vs. 27.8%; p = 0.007). After March 10 and March 22, the number of passengers actually increased in many stations, and “old” stations reported a greater percentage increase than “young” stations after March 22 (4.0% vs 1.1%; p = 0.007).

**Table 3:** Percentage change in the mean daily number of passengers and p-values for the mean differences between stations classified by age or purpose of visit The five days in headers represent the five important events: January 27 - the KCDC raises its infectious disease alert level from “Yellow” to “Orange”; February 20 - the first death due to COVID-19 in South Korea reported; February 29 - the number of new confirmed cases reaches a peak with 909 cases; March 10 - mass infection reported for the first time in Seoul; March 22 - the KCDC starts a campaign for aggressive social distancing Reference week: January 13-19; minimum week: March 2-8; KCDC: Korea Centers for Disease Control and Prevention

	Reference vs. minimum week		January 27		February 20		February 29		March 10		March 22	
	Percentage change	P-value	Percentage change	P-value	Percentage change	P-value	Percentage change	P-value	Percentage change	P-value	Percentage change	P-value
By age		0.551		0.135		0.860		0.253		0.664		0.007
Young	-46.3		-15.7		-23.8		-9.7		3.3		1.1	
Old	-49.2		-22.3		24.3		-5.6		2.3		4.0	
By purpose		0.021		0.003		0.007		0.393		0.556		0.604
Work	-36.2		-10.0		-16.2		-11.0		0.6		2.4	
Leisure	-51.6		-21.0		-27.8		-8.8		2.1		1.4	

## Discussion

The current study shows how the passengers of the Seoul Metropolitan Subway changed their behavior at different time points throughout the outbreak of COVID-19 from January to March 2020. By identifying 16 subway stations based on age or purpose of the visit of the passengers, this study attempts to capture the behavioral changes of various groups of subway passengers. As COVID-19 continues to spread around the world, much of the question have been focused on how far social distancing measures should be implemented at the cost of individual freedom and economic impact [12]. In this context, the current work captures the extent to which social distancing was implemented in Seoul without forceful government intervention.

Regarding subway usage in general, the most marked change was observed after February 20, when the first death due to COVID-19 was reported and the number of new cases began to increase at an alarming rate following the identification of a mass infection cluster in Daegu. An early report also suggests that the first report of death led to a peak in risk perception among the Chinese people in Hubei during the outbreak [13]. However, in contrast to the results of the survey in late March in South Korea where 88.7% of the respondents replied that they refrained from using public transport, the actual overall decrease in subway use was 40.6% at most [4]. Thus, the actual risk perception regarding COVID-19 among the people of Seoul may be lower than expected.

Particularly concerning is the fact that despite the first episode of mass infection in Seoul on March 10 and the announcement of an aggressive social distancing campaign on March 22, the number of passengers did not decrease but increased instead, albeit at a slow pace. Indeed, according to a regular bi-monthly survey of 1,000 respondents, the proportion of respondents who thought the spread of COVID-19 in South Korea was “very serious” decreased from 46% to 24% between the beginning of March and the end of March [14]. From March 9 onwards, the number of new cases of COVID-19 in South Korea has hovered around 100, with Seoul experiencing 10-20 new cases each day. While the reason for this sustained occurrence of new cases is likely multifactorial, the social distancing in Seoul has been less intense than that in Daegu, and this may partially explain why the reproduction number in Seoul did not reduce below one [15].

While it is difficult to assume a single defining characteristic for each station, the changes in stations classified by age or purpose of the visit showed some interesting patterns. For instance, the number of passengers in “old” stations increased compared to “young” stations after March 22. This is a worrisome change since, as of April 9, case fatality proportion in South Korea for COVID-19 patients aged 70-79 and 80 or greater was 8.7% and 21.1%, respectively, in comparison to 2% for all ages [16]. Conversely, while there were concerns that young people had low awareness for social distancing, the distinct peak on weekends usually seen in Hongik University station, a popular nightlife destination for young people, became markedly blunt, suggesting that a large proportion of young people had developed increased risk perception towards COVID-19 [17]. However, people may have increased the use of other modes of transport, so the decrease in subway passenger numbers may not fully capture the true extent of social distancing. Since people with COVID-19 are more likely to be asymptomatic and may transmit the virus unknowingly, social distancing for this age group should continue to be monitored and encouraged [18].

The observation that changes were more drastic and rapid in “leisure” stations than “work” stations suggests that while people tried to reduce non-essential travel when they could, reducing commute was not as manageable. In particular, Gasan Digital Complex station showed a less marked decrease in the number of passengers than other “work” stations, which may be due to the fact that manufacturing-based startup companies are common in Gasan Digital Complex, and thus they might have had less flexibility or resources to work from home [19]. Policymakers may prioritize identifying, monitoring, and supporting such companies that are less capable of practicing social distancing to reduce the risk of workplace transmission of COVID-19.

There are several limitations to this study. Buses are also a widely used mode of public transport in Seoul, and cars may have been used more frequently by people who wanted to avoid crowded subways. Hence, the number of subway passengers may not fully capture the extent of the proportion of people actually leaving their homes. While each of the 16 stations selected was assumed to have a defining characteristic, such as “old” or “work,” the composition of passengers even in these stations can be quite heterogeneous and inferring behavioral changes of specific population groups based on such characterization of stations should be done with caution. Despite these limitations, the current work presents an objective and insightful snapshot of the response to COVID-19 in Seoul that may guide policymakers and the general public in shaping their future response to the current pandemic.

## Conclusions

The current study shows how subway use in Seoul, South Korea, has changed during the COVID-19 outbreak between January 1 and March 31, 2020. The results suggest that while the level of risk perception regarding COVID-19 in Seoul increased sharply initially, it has consequently steadily decreased, which is worrying in the context of persistent emergence of new cases of COVID-19 in Seoul. The differing patterns of subway use among population groups classified by age or purpose of the visit may aid policymakers when attempting to deliver effective interventions to encourage and enforce social distancing as well as guide the general public in shaping their future response to the ongoing pandemic. Future studies are needed to assess the consequences of changing levels of risk perception and social distancing on the trajectory of COVID-19.
